# Spontaneous Bilateral Spermatic Vein Thrombosis: A Rare Clinical Presentation

**DOI:** 10.7759/cureus.20161

**Published:** 2021-12-04

**Authors:** Jouhar J Kolleri, Abdirahman M Abdirahman, Nabil Sherif Mahmood, Sushila Ladumor, Safa Hameed

**Affiliations:** 1 Clinical Imaging, Hamad Medical Corporation, Doha, QAT; 2 General Medicine, Employees' State Insurance Corporation Hospital, Kollam, IND

**Keywords:** testicular vein thrombosis, acute scrotum, bilateral spermatic vein thrombosis, ultrasound doppler, spermatic vein thrombosis

## Abstract

Cases of acute scrotum presenting to the emergency department are usually due to testicular torsion, complicated hernia, or epididymo-orchitis. Thrombosis of the spermatic vein is an uncommon entity and most of the cases reported to date are of unilateral involvement. Here, we present an extremely rare case of spontaneous bilateral spermatic vein thrombosis, which was diagnosed by a Doppler ultrasound of the testes. This article highlights its clinical presentation, radiological imaging, etiology, and management.

## Introduction

Spermatic vein thrombosis is an uncommon event and needs thorough evaluation for differential diagnosis. The acute scrotum is the presenting complaint in most cases [1]. Isolated bilateral spontaneous spermatic vein thrombosis is a rare disease, and, to the best of our knowledge, only two cases were reported in the literature to date [1,2]. Doppler ultrasound plays a significant role in the early and accurate diagnosis of this unusual clinical condition. We discuss a case of bilateral spermatic vein thrombosis, its diagnosis, etiology, and management.

## Case presentation

A 38-year-old gentleman came to the emergency department with complaints of sudden onset of bilateral scrotal pain and lower abdominal pain. There was no history of vomiting, dysuria, obvious trigger, or similar illness in the past. He had transurethral resection of the prostate (TURS) and aspiration of prostatic abscess six years ago. The patient was a smoker and was working as a laborer carrying heavy objects. On examination, his vitals were stable. Local examinations showed no scrotal swelling. Mild erythema was present over the scrotum. Testes were firm, non-tender, and without masses or lesions. Other systemic examinations were within the normal limit. Basic blood investigations and coagulation profiles were normal.

**Figure 1:**
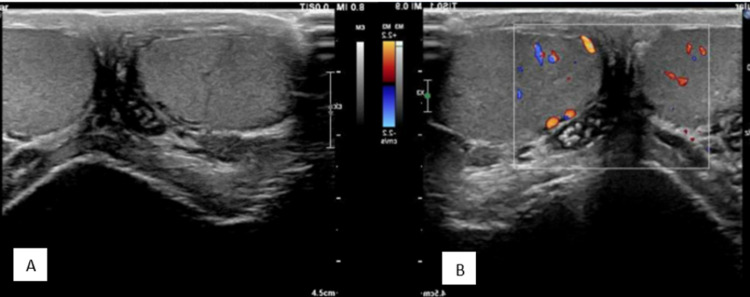
Doppler ultrasound of the testes showing (A) bilateral testes with normal echogenicity and (B) normal vascularization.

**Figure 2:**
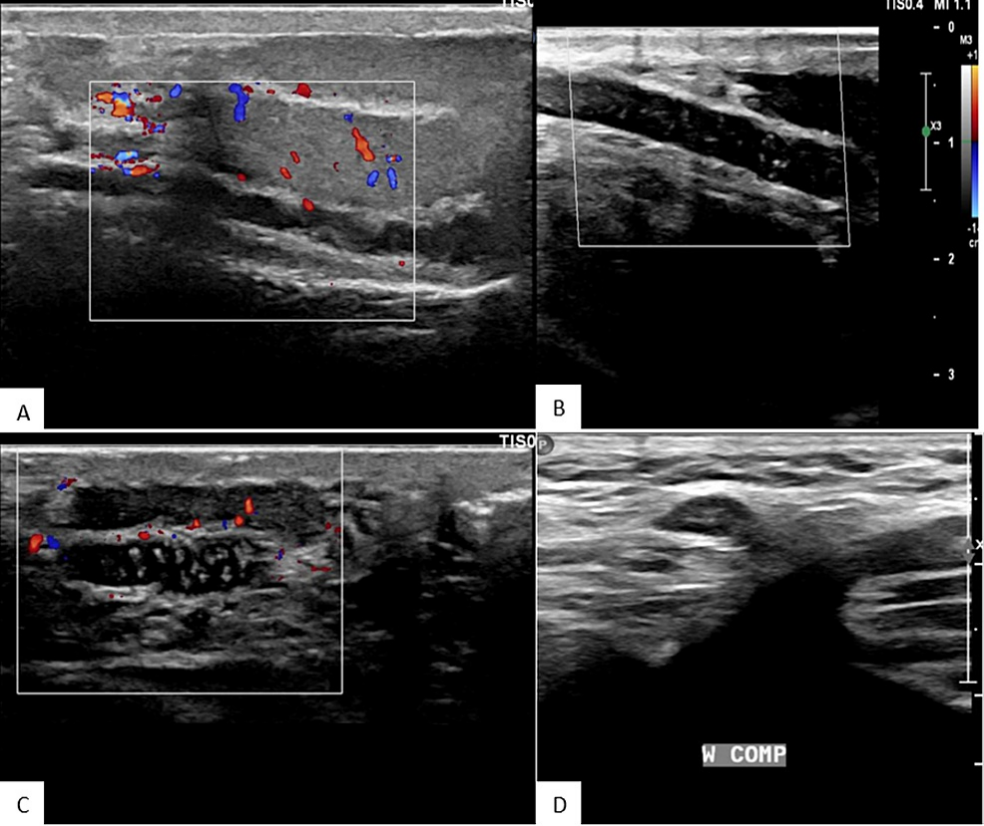
Doppler ultrasound of the testes showing non-compressible (A and B) right and (C and D) left spermatic veins with echogenic thrombus and no flow on Doppler.

A Doppler ultrasound of the testes was done from the emergency department to rule out testicular torsion, which showed bilateral testes with normal echogenicity and vascularity (Figure 1). Long segment intra-luminal echogenic thrombus was seen bilaterally extending from the spermatic cords to the inguinal veins with non-compressible and dilated spermatic cord veins, which gave an impression of bilateral spermatic cord venous thrombosis (Figure 2).

Computed tomography of the abdomen and pelvis showed no mass lesion. CT angiogram of the abdomen was done to assess the extent of spermatic vein thrombosis and to know if there is any other thrombus, which revealed patent major abdominal vessels with no evidence of filling defect. The patient was started on rivaroxaban 15 mg, twice daily for three weeks, followed by once a day for three to six months. The patient became symptomatically better after five days in the hospital and was discharged home with rivaroxaban tablets and anticoagulation clinic follow-up.

## Discussion

There are 42 reports of various thromboses of the pampiniform plexus, spermatic vein, and/or testicular vein found in the literature. Only two cases were found to be involving bilateral spermatic veins thrombosis and the most common site of occurrence was on the left side in other cases. Bilateral pampiniform plexus thrombosis reported by Kamel et al. was caused by protein C deficiency, whereas Bakshi described an idiopathic case mimicking incarcerated hernia [1,2]. The literature has described several etiologies for spermatic vein thrombosis, which include protein C deficiency, factor V Leiden mutation, underlying cancer, cardiac catheterization complications, inguinal region injury, and after suspected coronavirus disease 2019 (COVID-19) infection [1-7]. The nutcracker syndrome where there is compression of the left renal vein in the aortomesenteric space is also complicated by spermatic vein thrombosis in one study [8]. Retroperitoneal masses can cause venous blood flow obstruction and thrombosis, but the CT scan of our patient was normal.

All venous thromboses in some form or the other have been attributed to one of Virchow’s triads, which are endothelial damage, blood flow stasis, and hypercoagulability. Our patient had been working as a laborer carrying heavy objects for more than eight hours per day, which increased intra-abdominal pressure that might have reduced the spermatic blood flow causing stasis and thrombosis. Other factors were excluded as coagulation profiles were normal and there was no recent surgical history or trauma.

The low blood flow in the left testicular vein as it joins the left renal vein is the cause of left dominance of spermatic vein thrombosis, and any cause of decreased flow in the testicular vein such as genitourinary malignancy or conditions like nutcracker syndrome might lead to spermatic cord thrombosis [4]. Isolated right spermatic vein thrombosis reported in one study was associated with underlying factor V Leiden mutation [3].

The progression of spermatic vein thrombosis is variable; it can go away on its own, or it can be worsened by pulmonary embolism [6]. Conservative treatment, including anticoagulant and anti-inflammatory medications, as well as bed rest and scrotal support, should be the standard therapy [1]. According to the Canadian Association of Gastroenterology, anticoagulation therapy for three months is recommended when the cause of venous thrombosis is established [9]. Our patient was treated with rivaroxaban 15 mg, twice daily for three weeks, followed by once a day for three to six months. His hospital stay was uneventful and he was discharged home with rivaroxaban tablets for regular follow-up. Surgery was the norm until noninvasive imaging was readily accessible. With the introduction of imaging modalities, anticoagulation and anti-inflammatory medicines are being used to treat spermatic vein thromboses [7].

## Conclusions

Spontaneous bilateral spermatic venous thrombosis is an uncommon entity presenting as the acute scrotum. Therefore, a high threshold of suspicion is required as not to miss other causes of acute scrotal pain like torsion and incarcerated hernia, which will need surgical intervention. Doppler ultrasound is the first choice of investigation since it can also be used to assess other differentials. In patients with acute scrotal pain, it helps clinicians distinguish between those who need surgery and those who can benefit from conservative treatment alone. Conservative management is the initial treatment for bilateral spermatic vein thrombosis, which includes pain killers and anticoagulants.
